# 1-Benzoyl-4-(2-nitro­phen­yl)semicarbazide

**DOI:** 10.1107/S1600536810013048

**Published:** 2010-04-17

**Authors:** Ting Sun, Gong-Chun Li, Jing Li, Feng-Ling Yang

**Affiliations:** a College of Chemistry and Chemical Engineering, Xuchang University, Xuchang, Henan Province 461000, People’s Republic of China

## Abstract

The title compound, C_14_H_12_N_4_O_4_, was prepared by the reaction of 2-nitro­phenyl isocyanate with benzoyl­hydrazine. The dihedral angle between the rings is 71.49 (6)) Å. The mol­ecular conformation is stabilized by an intra­molecular N—H⋯O hydrogen bond, generating an *S*(6) ring. The crystal packing shows N—H⋯O hydrogen bonds.

## Related literature

For the bioactivity of urea derivatives, see: Yip *et al.* (1986 ▶); Liu *et al.* (2005 ▶).
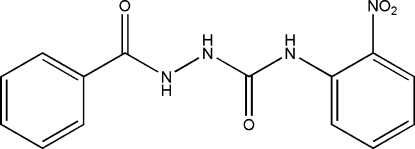


## Experimental

### 

#### Crystal data

C_14_H_12_N_4_O_4_
*M* *_r_* = 300.28
Monoclinic, 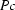

*a* = 4.655 (2) Å
*b* = 15.020 (7) Å
*c* = 10.005 (5) Å
β = 97.954 (7)°
*V* = 692.9 (5) Å^3^
*Z* = 2
Mo *K*α radiation
μ = 0.11 mm^−1^
*T* = 113 K
0.20 × 0.16 × 0.12 mm

#### Data collection

Rigaku Saturn 724 CCD area-detector diffractometer
Absorption correction: multi-scan (*CrystalClear*; Rigaku/MSC, 2005 ▶) *T*_min_ = 0.979, *T*_max_ = 0.987
5816 measured reflections
1652 independent reflections
1383 reflections with *I* > 2σ(*I*)
*R*_int_ = 0.033

#### Refinement

*R*[*F* ^2^ > 2σ(*F* ^2^)] = 0.028
*wR*(*F* ^2^) = 0.059
*S* = 0.96
1652 reflections
212 parameters
2 restraints
H atoms treated by a mixture of independent and constrained refinement
Δρ_max_ = 0.17 e Å^−3^
Δρ_min_ = −0.16 e Å^−3^

### 

Data collection: *CrystalClear* (Rigaku/MSC, 2005 ▶); cell refinement: *CrystalClear*; data reduction: *CrystalClear*; program(s) used to solve structure: *SHELXS97* (Sheldrick, 2008 ▶); program(s) used to refine structure: *SHELXL97* (Sheldrick, 2008 ▶); molecular graphics: *XP* (Sheldrick, 2008 ▶); software used to prepare material for publication: *SHELXL97*.

## Supplementary Material

Crystal structure: contains datablocks global, I. DOI: 10.1107/S1600536810013048/bt5244sup1.cif

Structure factors: contains datablocks I. DOI: 10.1107/S1600536810013048/bt5244Isup2.hkl

Additional supplementary materials:  crystallographic information; 3D view; checkCIF report

## Figures and Tables

**Table 1:** Hydrogen-bond geometry (Å, °)

*D*—H⋯*A*	*D*—H	H⋯*A*	*D*⋯*A*	*D*—H⋯*A*
N2—H2*A*⋯O3^i^	0.80 (2)	2.10 (2)	2.844 (2)	154.8 (18)
N3—H3*A*⋯O4^ii^	0.90 (2)	1.95 (2)	2.835 (2)	167 (2)
N1—H1⋯O2	0.83 (2)	2.13 (2)	2.640 (2)	119.8 (17)
N1—H1⋯O3^i^	0.83 (2)	2.32 (2)	2.987 (2)	138.7 (19)

Symmetry codes: (i) 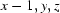; (ii) 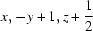.

**Fig. 1.**
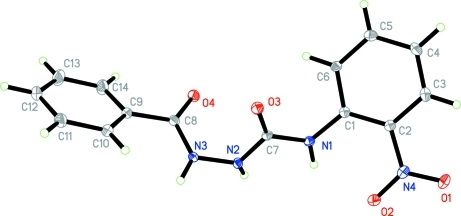
The asymmetric unit of the title compound with displacement ellipsoids drawn at the 30% probability level.

**Fig. 2.**
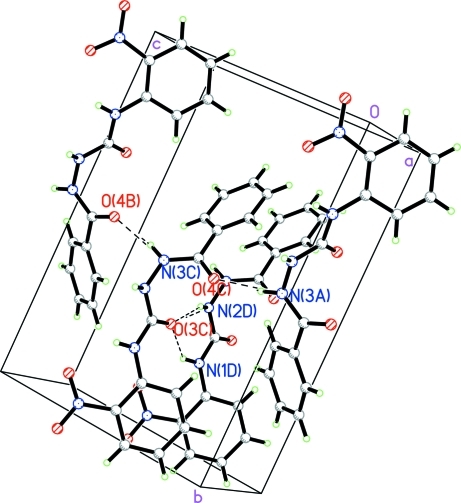
The packing diagram of the title compound. Intermolecular hydrogen bonds are shown as dashed lines.

## supplementary crystallographic information

### Comment

Urea derivates are very interesting reagents due to their useful properties and important medical and biological applications. For example, aryl urea derivates have been found to inhibit cell division as weedicides, and homologous aryl urea derivates have also been researched as antibacterial activity. Thidiazuron, a substituted heterocyclic urea compound, mimicked the effect of benzyladenine(BA) in the Ca^2+^ + cytokinin system and the IAA + cytokinin systems systems. (Yip   *et al.*, 1986). Recently, better activity of sectiona benzoyl urea derivates were reported. (Liu   *et al.*, 2005). In order to discover further biologically active urea compounds, the title compound was synthesized and its crystal structure was determined (Fig. 1). The molecular conformation is stabilized by an intramolecular N—H···O hydrogen bond. The crystal packing shows N—H···O hydrogen-bonds.  (Fig. 2).

### Experimental

2-nitrophenyl isocyanate(0.164 g, 1 mmol) and benzoyl hydrazine (0.136 g, 1 mmol) were milled  and mixed thoroughly in an agate mortar. Then the mixture  was put into a beaker and irradiated by microwave for 1 min. After the reaction was completed, the resulting mixture was dissolved in 95% ethanol   and filtrated. The products separated and were collected by filtration. The title compound was recrystallized from ethanol and single crystals   were obtained by slow evaporation.

### Refinement

In the absence of anomalous scatterers, the absolute structure could not be determined and, therefore, Friedel pairs were merged. All H atoms bonded to C atoms were placed in calculated positions, with C—H = 0.95 Å, and included in the   refinement using a riding model, with *U*_iso_(H) = 1.2U_eq_(C). H atoms bonded to N were freely refined.

### Crystal data

**Table:**

C_14_H_12_N_4_O_4_	*F*(000) = 312
*M**_r_* = 300.28	*D*_x_ = 1.439 Mg m^−^^3^
Monoclinic, *P**c*	Mo *K*α radiation, λ = 0.71073 Å
Hall symbol: P -2yc	Cell parameters from 2582 reflections
*a* = 4.655 (2) Å	θ = 1.4–27.9°
*b* = 15.020 (7) Å	µ = 0.11 mm^−^^1^
*c* = 10.005 (5) Å	*T* = 113 K
β = 97.954 (7)°	Prism, colorless
*V* = 692.9 (5) Å^3^	0.20 × 0.16 × 0.12 mm
*Z* = 2	

### Data collection

**Table:**

Rigaku Saturn 724 CCD area-detector diffractometer	1652 independent reflections
Radiation source: fine-focus sealed tube	1383 reflections with *I* > 2σ(*I*)
graphite	*R*_int_ = 0.033
Detector resolution: 14.68 pixels mm^-1^	θ_max_ = 27.9°, θ_min_ = 1.4°
ω and φ scans	*h* = −4→6
Absorption correction: multi-scan (*CrystalClear*; Rigaku/MSC, 2005)	*k* = −16→19
*T*_min_ = 0.979, *T*_max_ = 0.987	*l* = −13→12
5816 measured reflections	

### Refinement

**Table:**

Refinement on *F*^2^	Secondary atom site location: difference Fourier map
Least-squares matrix: full	Hydrogen site location: inferred from neighbouring sites
*R*[*F*^2^ > 2σ(*F*^2^)] = 0.028	H atoms treated by a mixture of independent and constrained refinement
*wR*(*F*^2^) = 0.059	*w* = 1/[σ^2^(*F*_o_^2^) + (0.0305*P*)^2^] where *P* = (*F*_o_^2^ + 2*F*_c_^2^)/3
*S* = 0.96	(Δ/σ)_max_ = 0.007
1652 reflections	Δρ_max_ = 0.17 e Å^−^^3^
212 parameters	Δρ_min_ = −0.16 e Å^−^^3^
2 restraints	Extinction correction: *SHELXL97* (Sheldrick, 2008), Fc^*^=kFc[1+0.001xFc^2^λ^3^/sin(2θ)]^-1/4^
Primary atom site location: structure-invariant direct methods	Extinction coefficient: 0.044 (4)

### Special details

**Table:**

Geometry. All esds (except the esd in the dihedral angle between two l.s. planes) are estimated using the full covariance matrix. The cell esds are taken into account individually in the estimation of esds in distances, angles and torsion angles; correlations between esds in cell parameters are only used when they are defined by crystal symmetry. An approximate (isotropic) treatment of cell esds is used for estimating esds involving l.s. planes.
Refinement. Refinement of *F*^2^ against ALL reflections. The weighted *R*-factor wR and goodness of fit *S* are based on *F*^2^, conventional *R*-factors *R* are based on *F*, with *F* set to zero for negative *F*^2^. The threshold expression of *F*^2^ > σ(*F*^2^) is used only for calculating *R*-factors(gt) etc. and is not relevant to the choice of reflections for refinement. *R*-factors based on *F*^2^ are statistically about twice as large as those based on *F*, and *R*- factors based on ALL data will be even larger.

### Fractional atomic coordinates and isotropic or equivalent isotropic displacement parameters (Å^2^)

**Table:**

	*x*	*y*	*z*	*U*_iso_*/*U*_eq_	
O1	0.4760 (3)	−0.01804 (9)	0.99297 (16)	0.0318 (4)	
O2	0.4185 (3)	0.12193 (9)	1.02923 (16)	0.0305 (4)	
O3	1.1178 (3)	0.32395 (8)	0.92954 (15)	0.0224 (3)	
O4	0.8286 (3)	0.48594 (9)	0.76324 (14)	0.0256 (3)	
N1	0.6951 (4)	0.24346 (11)	0.90518 (17)	0.0191 (4)	
H1	0.539 (5)	0.2410 (13)	0.936 (2)	0.021 (6)*	
N2	0.6976 (4)	0.38858 (10)	0.97176 (17)	0.0172 (3)	
H2A	0.523 (5)	0.3870 (12)	0.958 (2)	0.014 (5)*	
N3	0.8310 (3)	0.47099 (10)	0.98749 (17)	0.0183 (3)	
H3A	0.853 (5)	0.4899 (15)	1.073 (2)	0.031 (6)*	
N4	0.5259 (3)	0.06040 (10)	0.97133 (17)	0.0220 (4)	
C1	0.7987 (3)	0.16832 (12)	0.8453 (2)	0.0169 (4)	
C2	0.7208 (4)	0.08059 (13)	0.8735 (2)	0.0186 (4)	
C3	0.8283 (4)	0.00766 (13)	0.8095 (2)	0.0238 (5)	
H3	0.7730	−0.0509	0.8310	0.029*	
C4	1.0138 (4)	0.02023 (13)	0.7156 (2)	0.0244 (5)	
H4	1.0907	−0.0293	0.6734	0.029*	
C5	1.0872 (4)	0.10670 (13)	0.6832 (2)	0.0228 (4)	
H5	1.2108	0.1160	0.6165	0.027*	
C6	0.9833 (4)	0.17889 (12)	0.7465 (2)	0.0181 (4)	
H6	1.0377	0.2372	0.7229	0.022*	
C7	0.8559 (4)	0.31947 (12)	0.93330 (19)	0.0165 (4)	
C8	0.9025 (4)	0.51379 (12)	0.8784 (2)	0.0189 (4)	
C9	1.0848 (4)	0.59575 (12)	0.9075 (2)	0.0202 (4)	
C10	1.2623 (4)	0.60775 (13)	1.0298 (2)	0.0228 (4)	
H10	1.2638	0.5648	1.0996	0.027*	
C11	1.4379 (4)	0.68291 (13)	1.0494 (2)	0.0297 (5)	
H11	1.5598	0.6910	1.1328	0.036*	
C12	1.4367 (5)	0.74538 (13)	0.9493 (3)	0.0337 (5)	
H12	1.5576	0.7964	0.9636	0.040*	
C13	1.2588 (5)	0.73400 (15)	0.8273 (3)	0.0404 (6)	
H13	1.2560	0.7776	0.7583	0.048*	
C14	1.0851 (5)	0.65894 (14)	0.8064 (2)	0.0339 (5)	
H14	0.9656	0.6507	0.7224	0.041*	

### Atomic displacement parameters (Å^2^)

**Table:**

	*U*^11^	*U*^22^	*U*^33^	*U*^12^	*U*^13^	*U*^23^
O1	0.0371 (9)	0.0231 (8)	0.0370 (10)	−0.0064 (6)	0.0116 (7)	0.0067 (7)
O2	0.0351 (8)	0.0255 (8)	0.0349 (10)	0.0007 (7)	0.0189 (7)	0.0013 (7)
O3	0.0137 (6)	0.0229 (7)	0.0310 (8)	−0.0021 (5)	0.0050 (5)	−0.0033 (6)
O4	0.0360 (8)	0.0254 (7)	0.0149 (8)	−0.0060 (6)	0.0018 (6)	−0.0025 (6)
N1	0.0144 (8)	0.0190 (8)	0.0256 (10)	−0.0022 (6)	0.0089 (7)	−0.0017 (7)
N2	0.0137 (8)	0.0160 (8)	0.0223 (9)	−0.0024 (6)	0.0036 (6)	−0.0027 (7)
N3	0.0224 (8)	0.0174 (8)	0.0156 (9)	−0.0034 (6)	0.0045 (6)	−0.0038 (7)
N4	0.0203 (8)	0.0228 (9)	0.0224 (10)	−0.0023 (7)	0.0007 (7)	0.0040 (7)
C1	0.0114 (9)	0.0190 (9)	0.0196 (11)	−0.0001 (7)	−0.0002 (7)	−0.0003 (8)
C2	0.0158 (9)	0.0210 (9)	0.0185 (10)	−0.0010 (7)	0.0011 (7)	0.0002 (8)
C3	0.0229 (10)	0.0180 (9)	0.0295 (12)	0.0005 (8)	0.0002 (8)	−0.0009 (8)
C4	0.0233 (10)	0.0219 (10)	0.0281 (12)	0.0034 (8)	0.0038 (8)	−0.0063 (9)
C5	0.0191 (10)	0.0281 (11)	0.0217 (11)	−0.0005 (8)	0.0043 (8)	−0.0013 (9)
C6	0.0150 (8)	0.0198 (10)	0.0199 (11)	−0.0006 (7)	0.0033 (7)	0.0012 (8)
C7	0.0166 (9)	0.0201 (9)	0.0130 (10)	−0.0006 (7)	0.0024 (7)	0.0018 (7)
C8	0.0205 (10)	0.0182 (9)	0.0181 (10)	0.0011 (7)	0.0033 (7)	−0.0001 (8)
C9	0.0241 (10)	0.0174 (9)	0.0201 (10)	−0.0005 (8)	0.0066 (8)	−0.0016 (8)
C10	0.0245 (10)	0.0216 (10)	0.0228 (11)	−0.0028 (8)	0.0055 (8)	−0.0010 (8)
C11	0.0263 (11)	0.0307 (11)	0.0324 (13)	−0.0058 (9)	0.0049 (9)	−0.0086 (10)
C12	0.0414 (13)	0.0227 (11)	0.0395 (15)	−0.0104 (9)	0.0148 (10)	−0.0086 (10)
C13	0.0603 (16)	0.0267 (12)	0.0353 (16)	−0.0126 (11)	0.0107 (12)	0.0065 (10)
C14	0.0503 (13)	0.0265 (11)	0.0238 (13)	−0.0079 (10)	0.0012 (10)	0.0028 (9)

### Geometric parameters (Å, °)

**Table:**

O1—N4	1.226 (2)	C4—C5	1.393 (3)
O2—N4	1.233 (2)	C4—H4	0.9500
O3—C7	1.227 (2)	C5—C6	1.377 (3)
O4—C8	1.229 (2)	C5—H5	0.9500
N1—C7	1.373 (2)	C6—H6	0.9500
N1—C1	1.395 (2)	C8—C9	1.500 (3)
N1—H1	0.83 (2)	C9—C14	1.387 (3)
N2—C7	1.359 (2)	C9—C10	1.390 (3)
N2—N3	1.384 (2)	C10—C11	1.392 (3)
N2—H2A	0.80 (2)	C10—H10	0.9500
N3—C8	1.348 (3)	C11—C12	1.371 (3)
N3—H3A	0.90 (2)	C11—H11	0.9500
N4—C2	1.456 (3)	C12—C13	1.388 (3)
C1—C6	1.405 (3)	C12—H12	0.9500
C1—C2	1.406 (3)	C13—C14	1.386 (3)
C2—C3	1.396 (3)	C13—H13	0.9500
C3—C4	1.374 (3)	C14—H14	0.9500
C3—H3	0.9500		
			
C7—N1—C1	123.34 (16)	C5—C6—C1	121.51 (18)
C7—N1—H1	116.3 (14)	C5—C6—H6	119.2
C1—N1—H1	119.8 (14)	C1—C6—H6	119.2
C7—N2—N3	117.57 (16)	O3—C7—N2	123.16 (16)
C7—N2—H2A	120.0 (14)	O3—C7—N1	123.73 (17)
N3—N2—H2A	118.2 (13)	N2—C7—N1	113.05 (15)
C8—N3—N2	119.45 (16)	O4—C8—N3	121.89 (17)
C8—N3—H3A	128.0 (15)	O4—C8—C9	122.59 (17)
N2—N3—H3A	112.5 (15)	N3—C8—C9	115.49 (17)
O1—N4—O2	122.58 (17)	C14—C9—C10	119.50 (18)
O1—N4—C2	118.03 (17)	C14—C9—C8	118.53 (18)
O2—N4—C2	119.39 (15)	C10—C9—C8	121.90 (17)
N1—C1—C6	119.49 (16)	C9—C10—C11	119.69 (19)
N1—C1—C2	123.91 (16)	C9—C10—H10	120.2
C6—C1—C2	116.54 (16)	C11—C10—H10	120.2
C3—C2—C1	121.65 (18)	C12—C11—C10	120.6 (2)
C3—C2—N4	116.19 (16)	C12—C11—H11	119.7
C1—C2—N4	122.16 (16)	C10—C11—H11	119.7
C4—C3—C2	120.32 (19)	C11—C12—C13	120.0 (2)
C4—C3—H3	119.8	C11—C12—H12	120.0
C2—C3—H3	119.8	C13—C12—H12	120.0
C3—C4—C5	119.01 (19)	C14—C13—C12	119.8 (2)
C3—C4—H4	120.5	C14—C13—H13	120.1
C5—C4—H4	120.5	C12—C13—H13	120.1
C6—C5—C4	120.9 (2)	C13—C14—C9	120.4 (2)
C6—C5—H5	119.5	C13—C14—H14	119.8
C4—C5—H5	119.5	C9—C14—H14	119.8
			
C7—N2—N3—C8	67.4 (2)	N3—N2—C7—O3	10.0 (3)
C7—N1—C1—C6	−35.8 (3)	N3—N2—C7—N1	−172.79 (16)
C7—N1—C1—C2	146.84 (18)	C1—N1—C7—O3	−12.5 (3)
N1—C1—C2—C3	179.25 (18)	C1—N1—C7—N2	170.32 (17)
C6—C1—C2—C3	1.8 (2)	N2—N3—C8—O4	7.0 (3)
N1—C1—C2—N4	−1.2 (2)	N2—N3—C8—C9	−171.28 (16)
C6—C1—C2—N4	−178.62 (16)	O4—C8—C9—C14	23.4 (3)
O1—N4—C2—C3	1.8 (2)	N3—C8—C9—C14	−158.26 (18)
O2—N4—C2—C3	−178.46 (17)	O4—C8—C9—C10	−153.41 (19)
O1—N4—C2—C1	−177.80 (17)	N3—C8—C9—C10	24.9 (2)
O2—N4—C2—C1	2.0 (2)	C14—C9—C10—C11	0.0 (3)
C1—C2—C3—C4	−0.4 (3)	C8—C9—C10—C11	176.84 (17)
N4—C2—C3—C4	180.00 (16)	C9—C10—C11—C12	0.2 (3)
C2—C3—C4—C5	−1.4 (3)	C10—C11—C12—C13	0.1 (3)
C3—C4—C5—C6	1.8 (3)	C11—C12—C13—C14	−0.7 (4)
C4—C5—C6—C1	−0.4 (3)	C12—C13—C14—C9	1.0 (4)
N1—C1—C6—C5	−178.97 (17)	C10—C9—C14—C13	−0.6 (3)
C2—C1—C6—C5	−1.4 (3)	C8—C9—C14—C13	−177.6 (2)

### Hydrogen-bond geometry (Å, °)

**Table:**

*D*—H···*A*	*D*—H	H···*A*	*D*···*A*	*D*—H···*A*
N2—H2A···O3^i^	0.80 (2)	2.10 (2)	2.844 (2)	154.8 (18)
N3—H3A···O4^ii^	0.90 (2)	1.95 (2)	2.835 (2)	167 (2)
N1—H1···O2	0.83 (2)	2.13 (2)	2.640 (2)	119.8 (17)
N1—H1···O3^i^	0.83 (2)	2.32 (2)	2.987 (2)	138.7 (19)

Symmetry codes: (i) *x*−1, *y*, *z*; (ii) *x*, −*y*+1, *z*+1/2.
